# The Effect of Semantic Similarity on Learning Ambiguous Words in a Second Language: An Event-Related Potential Study

**DOI:** 10.3389/fpsyg.2020.01633

**Published:** 2020-07-14

**Authors:** Yuanyue Zhang, Yao Lu, Lijuan Liang, Baoguo Chen

**Affiliations:** ^1^Beijing Key Laboratory of Applied Experimental Psychology, Faculty of Psychology, Beijing Normal University, Beijing, China; ^2^Center for Linguistics and Applied Linguistics, School of English and Education, Guangdong University of Foreign Studies, Guangzhou, China

**Keywords:** second language, ambiguous word, semantic similarity, homonym, polysemy

## Abstract

Ambiguous words have multiple meanings. How these multiple meanings interact with each other during ambiguous word learning remains unclear. The current study adopted an event-related potentials (ERPs) technique to explore whether there is an interaction between two meanings when learning second language (L2) ambiguous words and how semantic similarity affects ambiguous word learning. In order to explore this issue, Chinese–English bilinguals were asked to learn pseudowords, which were paired with either two related new meanings (polysemes), two unrelated new meanings (homonyms), or one single new meaning (monosomies) over 2 consecutive days. ERP results revealed that learning the second meaning of a homonym induced a more negative N400 than the first meaning; learning the second meaning of a polyseme tended to produce a more positive late component (LPC) than the first meaning. These results indicate that the first meaning of homonyms may interfere with learning their second meaning. However, the first meaning of polysemous words may facilitate learning their second meaning. The current findings suggest that different mechanisms might be involved in learning L2 homonyms and polysemes.

## Introduction

Vocabulary is very important to acquire a new language. Therefore, a number of studies have been conducted to explore second language (L2) word learning (McLaughlin et al., 2004; Elgort, 2011; Elgort and Piasecki, 2014; Kapa and Colombo, 2014; Yang et al., 2015). However, most of these studies focused on learning words with one single meaning. In fact, words with more than one meaning/sense (thereafter, meaning) dominate the vocabulary in all languages.

Those words with more than one meaning are called ambiguous words. Based on the semantic relationship among meanings, ambiguous words can be categorized into at least two classes. Homonyms are one class of ambiguous words, and they have unrelated multiple meanings. For example, *bank* means *financial institution*, and it also means *land near the river*. Polysemes are another class of ambiguous words, and they have multiple related meanings. For example, *head* means *part of body*, *top, leader, et al*., which are all related to each other. Compared to unambiguous words, ambiguous words have multiple meanings which are semantically related or unrelated to some degree. Therefore, investigations into the learning mechanism of ambiguous words can reveal how these multiple meanings interact with each other. The present study aimed to explore this interactional mechanism during L2 ambiguous word learning as well as how the internal semantic similarity between the meanings of ambiguous words modulates this mechanism.

### Previous Studies on Ambiguous Word Learning

In Degani and Tokowicz (2010) study, native English speakers were asked to learn English–Dutch translation equivalents, which were equally separated into two groups: unambiguous words (i.e., each English word has one single Dutch meaning, e.g., science – wetenschap), and ambiguous words (i.e., each English word has two Dutch meanings, e.g., change – verandering, wisselgeld). The results of both translation recognition task and Dutch-to-English production task showed that it was harder to learn ambiguous words than unambiguous words regardless of the time when the task was performed, either immediately after learning or 1 week later. These results suggest there was a learning disadvantage for ambiguous words. This disadvantage was more obvious for words with ambiguity in form (i.e., form-ambiguous words like two Dutch synonyms corresponding to one English word) compared to words with ambiguity in meaning (i.e., meaning-ambiguous words like each Dutch translation corresponding to a different meaning of an ambiguous English word).

Based on the findings of Degani and Tokowicz (2010) and Degani et al. (2014) took a further step to explore the impact of meaning learning method on ambiguous word learning. Two methods were chosen: the two meanings of each ambiguous word were learned in consecutive trials in the same session (thereafter, learning together), and the two meanings of each ambiguous word were learned in separate trials in different sessions (thereafter, learning separately). The results of both translation recognition task and Dutch-to-English production task showed that translation-ambiguous words were more difficult to learn than translation-unambiguous words. Moreover, the learning effect was better in the condition of learning together than the condition of learning separately. This finding suggests that the way in which ambiguous words are learned affects their learning outcome. In the current study, the meanings of ambiguous words were learned sequentially one after the other, and this learning method may need to be taken into consideration when interpreting the results of this study.

In Rodd et al. (2012) participants were required to learn a new meaning which was artificially paired to a related or unrelated unambiguous word, resembling the process of learning an ambiguous word. They found that learning related new meanings was easier than learning unrelated new meanings, and this learning advantage persisted even 1 week later. This result suggests that it is easier for a new meaning to get integrated into the existing semantic network when it is related to the known meaning of an ambiguous word (Rodd et al., 2002).

In the study of Bracken et al. (2017) native English speakers learned novel German ambiguous words for three training cycles. Immediate and 1-week delayed translation recognition task were used to test the learning performance. Results showed participants were slower and less accurate in the 1-week delayed translation recognition task when the multiple meanings of ambiguous words were less related. According to the authors, semantic similarity effect on L2 ambiguous word learning stems from the difficulty in establishing one-to-many form-meaning mapping for ambiguous words with unrelated meanings compared to those with related meanings.

Lu et al. (2017) asked native Chinese speakers to learn English pseudowords, each of which was paired with two unrelated L1 meanings, over 4 consecutive days. Cross-language semantic relatedness judgment task was adopted to examine learning performance. Results showed that the late-learned second meaning was harder to learn than the first-learned first meaning, which was reflected by low accuracy and slow response in cross-language semantic relatedness judgement. Furthermore, inhibitory control ability, which was tested by the Stroop task, was significantly negatively related to the reaction time of judging the second meanings. However, no significant correlation of relatedness was found between inhibitory control ability and the reaction time of judging the first meanings. These results suggest that the new meaning of L2 ambiguous words is harder to learn than the prior meaning, and inhibition control ability positively predicts the learning performance for the new meaning of L2 ambiguous words.

In Zhang et al. (2018) study, Chinese learners of English were asked to learn familiar English words which were paired with a new related or unrelated Chinese meaning over 3 consecutive days. A test (translation recognition) was performed immediately after the learning phase each day. By comparing the learning speed of different meaning types, they found that semantic similarity impacted the learning of new meanings of familiar L2 words, that is, learning related new meanings was faster and easier than learning unrelated new meanings.

Although Zhang et al. (2018) has found that the L2 known meaning affects the late-learned meaning, the question how those multiple meanings interact with each other during L2 ambiguous word learning remains unclear, since Zhang et al. (2018) only explored the effect of a previously learned meaning on a late-learned meaning. Moreover, the learning and retrieving phases cannot be easily separated in their experimental design. Participants might finish learning the pairs on the first day and use retrieval strategy for the next 2 days, making it hard to determine whether the impacts of the known meaning on the late-learned meaning occur during the learning process indeed. Additionally, a new meaning was paired to a familiar L2 word, and the pair with unrelated meanings was highly artificial to the learners, which may lower learners’ motivation in learning the new meaning in the unrelated pairs. Therefore, a more sensitive technique and neutral learning material should be used to explore the interactive mechanism during L2 ambiguous word learning.

### The Current Study

In sum, we know little about how the multiple meanings interact with each other during L2 ambiguous word learning. The present study aimed to explore this interactional mechanism as well as how semantic similarity affects ambiguous word learning via the event-related potentials (ERPs) technique. Compared to behavioral experiments, the ERP technique is more sensitive at detecting online cognitive processes because of its high temporal resolution, and it has been widely used in the word learning field (Van Petten and Kutas, 1990; McLaughlin et al., 2004; Perfetti et al., 2005; Pu et al., 2016; Chen et al., 2017).

The N400 component is the most used index, and it usually occurs in the 300–500 ms time window after the stimulus is presented (Mestres-Missé et al., 2007; Balass et al., 2010). The amplitude of N400 usually represents the difficulty of semantic integration. For example, the more difficult it is for a new meaning to get integrated into semantic context, the greater the N400 amplitude is (Kutas and Hillyard, 1980; Hillyard and Kutas, 1983; Rolke et al., 2001; Proverbio et al., 2004; Lau et al., 2008; Kutas and Federmeier, 2011).

Previous research using ERPs has shown that L2 vocabulary acquisition trajectory can be recorded by the change of N400 amplitude. For example, McLaughlin et al. (2004) found that learners showed a semantic priming N400 effect after 63 h of instruction, in which the N400 amplitudes to target words which were related to prime words were smaller than those which were unrelated to prime words. Perfetti et al. (2005) found an N400 semantic priming effect of a learned word after a short-time training. Pu et al. (2016) asked native English (L1) speakers to learn 100 Spanish (L2) words for less than 2 h each day during 2 days through the association pairing of each Spanish word with its English translation counterpart. A backward translation recognition task (L2–L1) was administered after the learning phase. Participants’ ERP responses were recorded during the task. Results showed that L1 translation counterparts elicited smaller N400 component than unrelated L1 targets, suggesting that the N400 could reflect different L2 word learning performances even when less than 4 h were spent on the learning. Based on this finding, it is appropriate to use N400 as an index for L2 ambiguous word learning in the current study.

In the field of ambiguous word processing, Haro et al. (2017) explored the difference in processing mechanism between English ambiguous words and unambiguous words, using the ERP technique. The results of a lexical decision task showed that ambiguous words induced more negative N400 than unambiguous words, indicating that the N400 can be used to reflect semantic level process for word ambiguity. Therefore, the present study adopted the N400 component as an index to reflect L2 ambiguous word learning.

The second ERP index is the late positive component (LPC) (Rugg and Doyle, 1994). Previous studies on memory found that recognizing previously learned words (old words) induced larger LPC than recognizing new words (Rugg, 1995). The LPC related to the old/new effect is usually observed in the central-posterior area in the left hemisphere and peaks at approximately 600 ms (Wilding and Rugg, 1996). This component may reflect the encoding strength of memory (Paller et al., 1987; Paller and Wagner, 2002), episodic memory retrieval (Rugg and Curran, 2007), and controlled semantic retrieval (Martin et al., 2009). For example, previous studies about memory have found that the stimulus events remembered during the testing phase elicit larger LPC at the memory encoding stage, compared with the stimulus events forgotten during the testing phase. This phenomenon is known as the DM effect (difference due to memory) (Paller and Wagner, 2002; Van Strien et al., 2007; Danker et al., 2008). Johnson et al. (1998) further found that the LPC can be divided into three different subcomponents: the first subcomponent is mainly located in the left medial-frontal area with a latency of 400–490 ms; the second subcomponent which is related to explicit memory is mainly located in the left parietal-occipital area with a latency of 500–700 ms; the third subcomponent which may be related to the access of contextual information is mainly located in the right central-frontal area with an onset between 500 and 590 ms. Peters and Daum (2009) suggested that the parietal LPC reflects more general memory retrieval, whereas the frontal LPC reflects episodic memory retrieval. In general, these findings indicate that LPC might be related to the process of memory’s encoding, consolidation and retrieval. Based on these studies, we would also focus on the LPC in the preset study, because L2 ambiguous word learning implicates the processes of encoding, consolidation, and retrieval of multiple meanings. If the meanings are different in the intensity of encoding, they may induce different LPCs.

Specifically, we selected Chinese–English bilinguals as our participants. To avoid the interference of past learning experience, we used pseudowords as learning materials. We paired one or two Chinese meanings to each English pseudoword. There were three types of pseudowords: pseudowords paired with one single meaning, which are parallel to monosemes or unambiguous words; pseudowords paired with two unrelated meanings, which are parallel to homonyms; and pseudowords paired with two related meanings, which are parallel to polysemes. Participants were required to learn the meaning/meanings of these pseudowords for 3 consecutive days. The learning process was recorded with EEG. On day 1 and day 2, the unambiguous words and the first meaning of homonyms and polysemous words were learned. The second meaning of homonyms and polysemous words were learned on the day 2 and day 3.

There still lacks a definite theoretical formulation which can be used to predict the interaction among the multiple meanings of L2 ambiguous words. In the field of word recognition, some studies have found differences between processing unambiguous words and ambiguous words (Klepousniotou and Baum, 2007; Klepousniotou et al., 2012; Haro and Ferré, 2018; Haro et al., 2019). For example, it takes less time to recognize ambiguous words than unambiguous words (Borowsky and Masson, 1996; Hino and Lupker, 1996; Haro and Ferré, 2018; Haro et al., 2019). Moreover, some studies further found homonym disadvantage and polysemy advantage compared to processing unambiguous words (Rodd et al., 2002; Beretta et al., 2005). The homonym disadvantage and polysemy advantage might be accounted for by one kind of model of ambiguous word processing developed from the view of parallel distributed processing, which suggests that separate semantic features are mapped onto the same word form for homonyms, whereas shared semantic features are mapped onto the same word form for polysemes. Therefore, when accessing a particular meaning of ambiguous words, there is competition among the multiple unrelated meanings of homonyms, which delays recognition, but a facilitation among the multiple related meanings of polysemes, which speeds up recognition (Klepousniotou, 2002; Rodd et al., 2004; Klepousniotou and Baum, 2007).

Based on the above reasoning, we predicted that there may be interaction effects between the first- and late-learned meanings, as reflected by ERP amplitude (for example, N400 and LPC) differences between the first and second meaning of ambiguous words. We also predicted that semantic similarity influences the interaction among the multiple meanings of L2 ambiguous words, as reflected by a mediation of semantic similarity in the ERP amplitude change for first- and late-learned meanings.

## Methods

### Participants

Twenty-three (10 males) right-handed, native Chinese speakers participated in the study (mean age 21.34 ± 2.78 years). They were recruited from several universities in Beijing and received monetary compensation for their participation. All participants had normal or corrected-to-normal vision. The ethical approval of all experimental procedure was obtained from the Committee of Protection of Subjects at Beijing Normal University. All participants provided the written informed consent form before the experiment.

The participants had begun to learn English at a mean age of 10.26 ± 1.69 years old. The Oxford Placement Test (OPT, maximum score = 50, includes 25 multiple choice questions and a cloze test) and self-rating scale (6-point scale, 1 for L2 skills being much worse than L1 skills, 6 for L2 skills being just as good as L1 skills) were used to assess the English proficiency. The self-rating scores of L2 skills were 3.21 ± 1.27 for listening, 3.13 ± 1.01 for speaking, 2.82 ± 0.93 for reading, and 2.78 ± 0.99 for writing. The OPT score was 39 ± 3.91. According to the self-rating and OPT score, the participants we recruited were late unbalanced Chinese–English bilinguals with intermediate English proficiency.

### Material

Participants were required to learn 105 English pseudowords. These pseudowords were all two-syllable and 6–8 letters in length and were created using Wuggy, a pseudoword generator (Keuleers and Brysbaert, 2010).

The 105 pseudowords were equally split into three sets. Thirty-five pseudowords were paired with one meaning 0in Chinese to constitute monosomies (unambiguous words), e.g., rebube-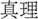 (truth) (hereafter, M). Thirty-five pseudowords were paired with two unrelated meanings in Chinese to constitute homonyms, e.g., nalpew-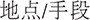 (site/method) (thereafter, H1 stands for the first meaning of homonyms, and H2 stands for the second meaning of homonyms, see Supplementary Table 2). Thirty-five pseudowords were paired with two related meanings in Chinese to constitute polysemes, e.g., soctur-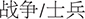 (war/soldier) (thereafter, P1 stands for the first meaning of polysemes, and P2 stands for the second meaning of polysemes, see Supplementary Table 1). The first and second meanings (H1, H2; P1, P2) were randomly assigned and kept constant for all participants.

The word length of the three sets of pseudowords (monosomies: 6.11 ± 0.10, polysemes: 6.11 ± 0.10, and homonyms: 6.03 ± 0.11) were matched, and the ANOVA results showed no significant difference among the three sets of pseudowords in word length [*F*(2,68) = 0.20, *p* = 0.82]. Moreover, we computed the average Orthographic Levenshtein Distance between the non-word and its 20 most similar words in the lexicon (OLD20, Yarkoni et al., 2008). The bigger the value of OLD20, the less the non-words look like to real words. The ANOVA results showed no significant difference among the three sets of pseudowords in OLD20 [monosomies: 2.46 ± 0.06, polysemes: 2.49 ± 0.06, and homonyms: 2.48 ± 0.06; *F*(2,68) = 0.04, *p* = 0.96]. The bigram frequency among the three sets of pseudowords was also not significant [monosomies: 655 ± 450, polysemes: 735 ± 509, and homonyms: 558 ± 355; *F*(2,68) = 1.63, *p* = 0.20].

Twenty-two additional participants from the same background were recruited to rate the similarity of the two meanings of the ambiguous words, in a scale ranging from 1.00 (unrelated) to 7.00 (related). The semantic relatedness score of homonym pairs (1.47 ± 0.23) was significantly smaller than that of polyseme pairs [6.21 ± 0.34, *t*(34) = 69.85, *p* < 0.001, *Cohen’s d* = 11.81]. The Chinese meanings were all two-character words which were chosen from the Modern Chinese Frequency Dictionary. The same 22 participants as those rating semantic similarity evaluated the familiarity of the Chinese meanings on a 7- point scale (1 for the least familiar, and 7 for the most familiar). The concreteness and imageability of those Chinese words were calculated for their English translation counterparts based on the MRC Psycholinguistic Database (Version 2.00; Wilson, 1988). Several repeated one-way ANOVAs were conducted among the five sets of meaning (M, H1, H2, P1, and P2) on familiarity, frequency (per million, Modern Chinese Frequency) stroke number concreteness and imageability. No significant differences were observed for any of the lexical properties, for familiarity [*F*(4,136) = 1.27, *p* = 0.28], for concreteness [*F*(4,136) = 0.89, *p* = 0.47], for imagery [*F*(4,136) = 0.86, *p* = 0.49], for frequency [per million, *F*(4,136) = 0.27, *p* = 0.90], or for stroke number [*F*(4,136) = 0.43, *p* = 0.78]. The lexical properties of the five sets of Chinese meanings are presented in Table 1.

**TABLE 1:** The lexical properties of the five sets of Chinese meanings, mean (SD).

Meaning type	Familiarity	Concreteness	Imagery	Frequency	Stroke number
M	6.58 (0.27)	403 (97)	445 (84)	52 (79)	17.20 (4.59)
P1	6.66 (0.22)	419 (120)	478 (91)	62 (103)	16.69 (3.45)
P2	6.69 (0.17)	392 (101)	455 (94)	48 (59)	16.17 (5.32)
H1	6.60 (0.20)	418 (112)	441 (108)	39 (144)	17.17 (4.68)
H2	6.43 (0.89)	437 (120)	448 (104)	45 (67)	16.06 (4.35)

*M, monosemes; P1, the first meaning of polysemy; P2, the second meaning of polysemy; H1, the first meaning of homonym; H2, the second meaning of homonym.*

### Design

We used 5 (meaning type: M, P1, P2, H1, and H2) × 2 (learning session: session A, session B) within-subject design.

### Procedure

Participants were required to learn 105 English pseudowords paired with Chinese meaning/meanings over 3 consecutive days. The arrangement of learning times and procedure are shown in Table 2. There were two learning sessions for each type of meaning. The monosomies (M) and the first meaning of polysemes (P1) and homonyms (H1) were learned on the first day (session A) and the second day (session B). The second meaning of polysemes (P2) and homonyms (H2) were learned on the second day (session A) and the third day (session B). Therefore, session A means first encounter and session B means second encounter. To be noted, P2 and H2 were learned first, followed by the M, P1, and H1 on Day 2. Participants learned each meaning repeatedly, 6 times in total over 2 consecutive days. Specifically, M, P1, and H1 were learned three times per day on day 1 and day 2. P2 and H2 were learned three times per day on day 2 and day 3 (see Table 2). In sum, in session A, all the meanings were learned for the first three times; in session B, all the meanings were learned for the second three times. Immediately after each learning phase, a 4-to-1 forced choice task was adopted to test the learning outcome.

**TABLE 2:** The learning times and learning procedure for each type of meaning.

	Day 1	Day 2	Day 3
Session A (1–3 times)	M P1 H1	P2 H2	
Session B (4–6 times)		M P1 H1	P2 H2

*M, monosomies; P1, the first meaning of polysemy; P2, the second meaning of polysemy; H1, the first meaning of homonym; H2, the second meaning of homonym.*

In order to make sure the word form of pseudowords would not add to the difficulty of meaning learning, a word list was sent to the participants 3 days earlier before the formal experiment. The word list included 105 pseudowords (35 pseudowords from each word type: monosomies, polysemes, and homonyms) with no meaning illustrations. Participants were required to get familiar with the word form of pseudowords during these 3 days. Then they had to perform a word form recognition task first to test their familiarity with the word form of the pseudowords prior to the formal experiment. This task required the participants to recognize the pseudowords they had learned among 210 words, half of which were target pseudowords and the other half were totally new pseudowords. Only those participant whose accuracy reached 90% could enter the formal experiment.

### Learning Task

**FIGURE 1:**
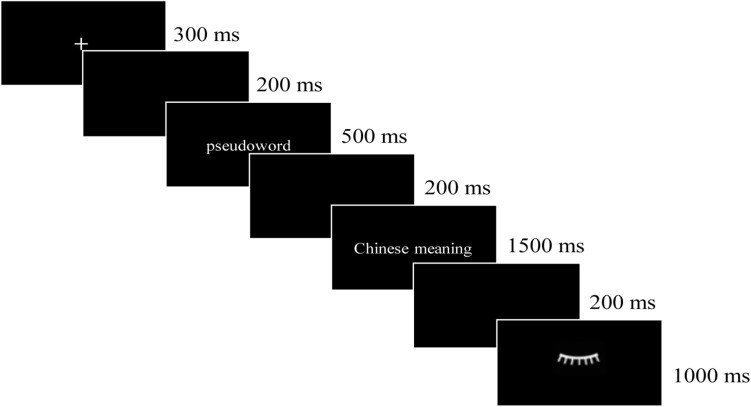
The procedure of learning.

The learning tasks were presented by E-prime 2.0, and the electroencephalogram (EEG) signal during learning was recorded. In each trial (see Figure 1), a fixation point (+) lasted for 300 ms, and then the English pseudoword was presented for 500 ms. After a 200 ms blank, the Chinese meaning(s) was/were presented for 1,500 ms, upon which the participants were required to memorize the English pseudowords and their corresponding Chinese meanings. After this, a blank screen was shown for 200 ms, and then an eyelash image was shown for 1000 ms, which reminds participants to relax their eyes. On the first day, each of the three meaning types (i.e., M, P1, and H1) was learned for three times, and that amounted to 315 trials in total (3 × 3 × 35). On the second day, the P2 and H2 were learned first and then the M, P1, and H1 were learned. Each of the five meaning types was learned for three times, and that amounted to 525 trials (5 × 3 × 35). On the third day, only the P2 and H2 were learned, and each of them was learned for three times, so there were 210 trials in total (2 × 3 × 35). All the words were pseudo-randomized during the learning phase to avoid the repetition of a same stimulus within 5 trials. Rest was allowed during the learning phase. Ten practice trials were presented before the formal learning experiment.

### 4-to-1 Forced Choice Task

After learning each day, we used a 4-to-1 forced choice task to check the learning outcome. The procedure was as follows: a fixation point lasted for 300 ms and was followed by a blank screen for 200 ms. Then the pseudowords that had been learned and four Chinese meaning choices were shown at the same time for 6,000 ms. Participants were required to choose the right Chinese meaning by pressing a number key (1, 2, 3, or 4 corresponding to the choice shown on the screen from left to right). A blank screen was shown for 200 ms after the key was pressed. One hundred and five trials were presented on the first learning day (lasting approximately 15 min), 175 trials were included on the second learning day (lasting approximately 25 min), and 70 trials were presented on the third learning day (lasting approximately 10 min). The order of trials was pseudo-randomized to prevent the same type of meaning being repeatedly shown in two consecutive trials. Twenty practice trials were used to familiarize the participants with the number keys.

### EEG Signal Recording and Pre-processing

During the learning task, the EEG signals were recorded from 64 Ag/AgCl electrodes positioned according to the extended 10–20 system. All scalp electrodes were referenced online to the right mastoid (M2) and re-referenced offline to the average of left and right mastoids (M1 and M2). The vertical eye movements were recorded by electrodes placed on the supra- and infra-orbital ridges of the left eye (VEOG), and the horizontal eye movements were recorded by electrodes placed on the outer canthi of the left and the right eyes (HEOG). Impedances were kept below 5 KΩ.

The EEG data were recorded using the NeuroScan 4.5 with a sampling rate of 500 Hz and filtered online with a bandpass between 0.05 and 100 Hz. Continuous recordings were segmented into epochs ranging from −200 to 800 ms relative to the onset of each Chinese word. Baseline correction was performed in reference to pre-stimulus activity (−200 to 0 ms). Trials were rejected if the amplitude on any channel exceeded ±100 μV. Average ERPs time-locked to the onset of each Chinese word were calculated for each condition by averaging across the correct response trials of the same type from −200 to 800 ms.

## Results

### Behavioral Data

Mixed-effects model (Baayen et al., 2008) was used in the data analysis with R Project for Statistical Computing using the lme4 package (Baayen et al., 2008). Accuracy data were analyzed with logistic regression after log transformation, and RT data were analyzed with linear regression. By-subject and by-item intercepts were included as random effects. If model comparisons showed a significant contribution and the models converged, a by-subject or by-item slope would be added. Fixed effects in the models included meaning type, learning session, and their interaction. The fixed effects were treatment-coded, that is, the reference level of meaning type was M and all the other meaning types, H1, H2, P1, and P2, were compared to M. Session A served as the reference level of learning session. The full model was compared to sub-models with (i) the fixed effect of meaning type removed (ii) the fixed effect of learning session removed and (iii) the interaction between meaning type and learning session removed. When a significant fixed effect was found, Tukey test was used for pairwise comparison, and Bonferroni-adjusted *p*-values were reported.

The accuracy data of 4-to-1 forced choice task was shown in Table 3. The LME model was constructed for accuracy data, with meaning type (M, H1, H2, P1, and P2), learning session (session A and session B) and their interaction as fixed effects, and with by-participant and by-item intercepts as random effects. The results showed that the accuracy for P2 was higher than for the other four types of meaning: M (*z* = 5.20, *p* < 0.001), P1 (*z* = 4.32, *p* < 0.01), H1 (*z* = 4.77, *p* < 0.001), and H2 (*z* = 4.34, *p* < 0.001). The differences of the other four meaning types were not significant (*p*s > 0.1). The accuracy of session B was significantly higher than that of session A (*z* = 8.13, *p* < 0.001). The interaction of learning type and learning session was not significant (χ^2^ = 7.43, *p* = 0.11).

**TABLE 3:** The accuracy data (%) and RT data (ms) of 4-to-1 forced choice task: mean (SD).

	M	P1	P2	H1	H2
**Session A**					
Accuracy	56 (18)	60 (18)	75 (15)	58 (17)	60 (22)
RT	3255 (447)	3210 (475)	2757 (562)	3256 (447)	3281 (637)
**Session B**					
Accuracy	74 (18)	78 (17)	87 (12)	76 (19)	83 (19)
RT	2662 (579)	2572 (579)	2241 (479)	2733 (555)	2519 (483)

*M, monosomies; P1, the first meaning of polysemy; P2, the second meaning of polysemy; H1, the first meaning of homonym; H2, the second meaning of homonym.*

For the response time (RT), only correct trials were included in the analysis. For each participant, the RTs beyond Mean ± 3 SD were excluded (2.23%). The linear mixed-effects model was constructed with meaning type (M, H1, H2, P1, and P2), learning session (session A and session B) and their interaction as fixed effects, and with by-participant and by-item intercepts as random effects. This was the same as the model constructed for the accuracy data.

The results showed that the interaction of meaning type and learning session was significant (χ^2^ = 24.29, *p* < 0.001). In session A, the RT for P2 was faster than that of the other four types of meaning: M (*z* = −5.06, *p* < 0.001), P1 (*z* = −5.051, *p* < 0.01), H1 (*z* = −5.62, *p* < 0.001), and H2 (*z* = −6.34, *p* < 0.001). The differences of the other four meaning types were not significant (*p*s > 0.1). In session B, the RT for P2 was also faster than that of the other four types of meaning: M (*z* = −3.86, *p* < 0.05), P1 (*z* = −2.77, *p* < 0.05), H1 (*z* = −4.44, *p* < 0.001), and H2 (*z* = −2.75, *p* < 0.001). The differences of the other four meaning types were not significant (*p*s > 0.1).

In sum, the behavioral data showed faster and more accurate response in recognizing P2 relative to the other four types of meaning regardless of learning session.

### ERP Data

One participant was removed due to few trials being usable after rejecting the artifacts (<56%). Thus, the data of 22 participants was included in statistical analysis. The average number of trails for each condition accounted for 90% of the total number of trails after removing the artifact. Mean amplitudes were computed between 300 and 500 ms for the N400 and 470–770 ms for the LPC upon the onset of the Chinese meaning.

After visual inspection of the current data, six electrodes for N400 were chosen from the central line and right hemisphere: FZ, F4, CZ, C4, PZ, and P4. Previous studies found that typical N400 effects are usually observed in the central-parietal area (Hillyard and Kutas, 1983; Brandeis et al., 1995). Therefore, six electrodes in the central-parietal area (CZ, C1, C2, CPZ, CP1, and CP2) were also selected for further ANOVA analysis. The time window for the LPC was 470–770 ms. Nine electrode sites were chosen: F3, FZ, F4, C3, CZ, C4, P3, PZ, and P4 (Finnigan et al., 2002). Greenhouse–Geisser-adjusted *p*-values were reported.

The way the ERP data were analyzed was different from that of the 4-to-1 forced choice task. As behaviroal data reflects the final outcome of learning, learning session was included in behavioral data analysis to guarantee a similar learning depth for the different meaning types to be compared. However, the ERP data reflects the real-time learning process. Therefore, we run separate analyses for Session A and Session B in order to investigate whether any interactions exist between the meanings during the learning process of ambiguous pseudowords (See Table 2 for the detailed learning arrangement). In Session A, the meanings M, H1, and P1 were learned 1 day earlier than H2 and P2, so the analysis would reveal whether there were any impacts from the first meaning on the second meaning or not. If yes, the ERP amplitudes of H2 and P2 would differ from those of M, H1, and P1. If not, the ERP amplitudes of the 5 types of meaning would be similar.

On day 2, the meanings H2 and P2 were learned prior to M, H1, and P1, so further analysis on M, H1, and P1 in Session B would reveal whether there were any impacts from the second meaning on the first meaning or not. If yes, the learning outcome of P1 and H1 would differ from that of M. If not, the learning outcome of P1, H1, and M would be similar. Therefore, a comparison among M, H1, and P1 in Session B would reveal the possible impact from the second meaning learning on the first meaning.

In addition, further analysis on H2 and P2 in session B would reveal participants’ learning performance on Day 3 when they only learned these meaning types.

### Session A

#### N400 (300–500 ms)

**FIGURE 2:**
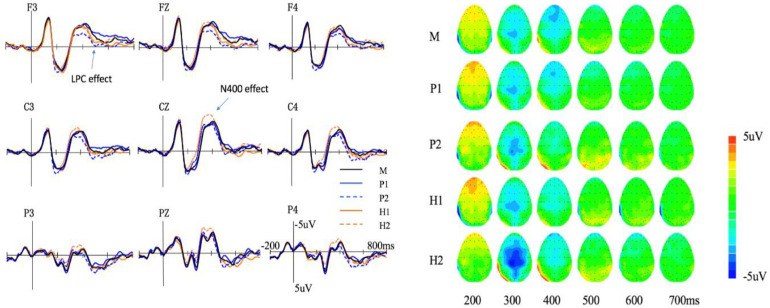
Event-related potentials (ERP) **(left)** and topography **(right)** of the five meaning types in session A. M, monosemes; P1, the first meaning of polysemy; P2, the second meaning of polysemy; H1, the first meaning of homonym; H2, the second meaning of homonym.

The average amplitude of the N400 of each meaning type is shown in Figure 2. A 5 (meaning type: M, H1, H2, P1, and P2) × 2 (hemisphere: right and central) repeated measures ANOVA was conducted. Results showed a significant main effect of meaning type [*F*(4,84) = 3.60, *p* < 0.01, $\eta_{\text{p}}^{2}$ = 0.14]. *Post hoc* analysis found that the N400 amplitude of H2 (−2.35 ± 0.40 μV) was more negative than that of P2 (−1.49 ± 0.42 μV) (*p* < 0.001, *Cohen’s d* = 0.88) and a marginally significant difference was found between the N400 amplitude of H2 and H1 (−1.22 ± 0.56 μV) (*p* = 0.07, *Cohen’s d* = 2.98). The main effect of hemisphere was significant [*F*(1,21) = 44.42, *p* < 0.001, $\eta_{\text{p}}^{2}$ = 0.67], and the N400 amplitude in the central area (−2.16 ± 0.47 μV) was more negative than that in the right hemisphere (−1.17 ± 0.40 μV). The interaction between meaning type and hemisphere was not significant [*F*(4,84) = 0.86, *p* > 0.1, $\eta_{\text{p}}^{2}$ = 0.04].

For the six electrodes in the central-parietal area (C1, CZ, C2, CP1, CPZ, and CP2), the ANOVA result showed a significant main effect of meaning type [*F*(8,168) = 2.19, *p* < 0.05, $\eta_{\text{p}}^{2}$ = 0.09]. Further analysis found that the amplitude of H2 (−2.87 ± 0.55 μV) was more negative than that of H1 (−1.65 ± 0.58μV, *p* = 0.02, *Cohen’s d* = 3.30) and P2 (−1.89 ± 0.56 μV, *p* < 0.001, *Cohen’s d* = 5.49). The differences among the other meaning types were not significant (*p*s > 0.1).

#### LPC (470–770 ms)

The average amplitude of the LPC of each meaning type is shown in Figure 2. A 5 (meaning type: M, H1, H2, P1, and P2) × 2 (hemisphere: left, central, and right) repeated measures ANOVA was conducted. Results showed the main effect of meaning type was not significant [*F*(4,84) = 1.90, *p* = 0.11, $\eta_{\text{p}}^{2}$ = 0.08]. The main effect of hemisphere was significant [*F*(1,21) = 7.04, *p* = 0.015, $\eta_{\text{p}}^{2}$ = 0.25], and the LPC amplitude in the left hemisphere (−0.20 ± 0.28 μV) was more negative than that in the right hemisphere (0.27 ± 0.25 μV). The interaction between meaning type and hemisphere was not significant [*F*(4,84) = 1.08, *p* > 0.1].

However, further analysis found a marginal difference (*p* = 0.06, *Cohen’s d* = 3.05) between P2 (−0.39 ± 0.32 μV) and P1 (−1.58 ± 0.37 μV) at the electrodes of the frontal area (F3, FZ, and F4). The second meaning of a polyseme tended to produce a more positive LPC than the first meaning. The differences were not significant at the other electrodes of the parietal (P3, PZ, and P4) and central areas (C3, CZ, and C4) (*p*s > 0.1).

In summary, in Session A, the N400 amplitude of H2 was more negative than that of H1, but no significant difference was found between P1 and P2. Moreover, the N400 amplitude of H2 was also more negative than that of P2. The LPC amplitude of P2 tended to be more positive than that of P1 in the frontal area.

### Session B

**FIGURE 3:**
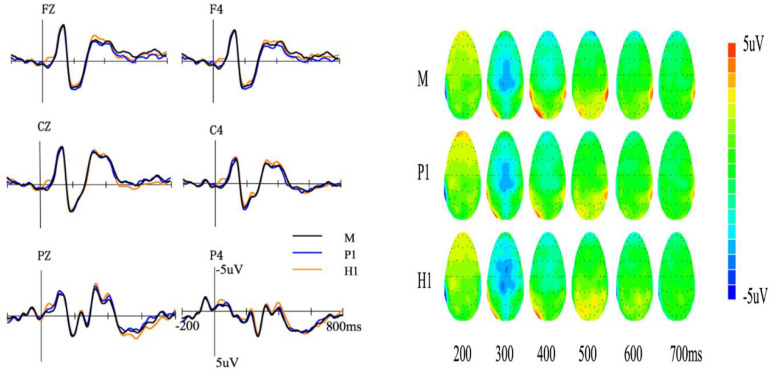
Event-related potentials **(left)** and topography **(right)** of the three meaning types in session B. M, monosomies; P1, the first meaning of polysemy; P2, the second meaning of polysemy; H1, the first meaning of homonym; H2, the second meaning of homonym.

In session B, we did not find any LPC differences, so we focused on the analysis of the N400. The average amplitudes of the N400 (300–500 ms) for the first meaning of ambiguous words and monosomies are shown in Figure 3. We conducted a 3 (meaning type: M, H1, and P1) × 2 (hemisphere: central and right) repeated measures ANOVA. Results showed no significant main effect of meaning type [*F*(2,42) = 0.83, *p* = 0.44, $\eta_{\text{p}}^{2}$ = 0.04]. Other main effects and their interaction were not significant (*p*s >0.1).

**FIGURE 4:**
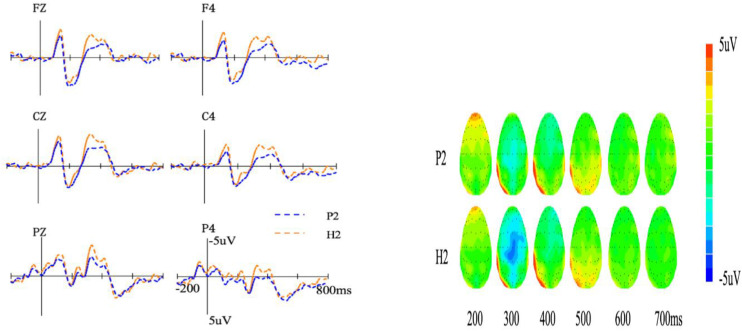
Event-related potentials **(left)** and topography **(right)** of the two meaning types in session B. P2, the second meaning of polysemy; H2, the second meaning of homonym.

We further analyzed the ERP data of H2 and P2 obtained on Day 3 when participants only learned these meaning types. The average amplitude of the N400 (300–500 ms) for the second meaning of ambiguous words is shown in Figure 4. We conducted a 3 (meaning type: H2 and P2) × 2 (hemisphere: central and right) repeated measures ANOVA. Results showed a significant main effect of meaning type [*F*(1,21) = 11.72, *p* < 0.01, $\eta_{\text{p}}^{2}$ = 0.35], and the N400 amplitude of H2 (−1.47 ± 0.41 μV) was more negative than that of P2 (−0.70 ± 0.40 μV). The interaction between meaning type and hemisphere was significant [*F*(1,21) = 7.78, *p* < 0.01, $\eta_{\text{p}}^{2}$ = 0.27]. Further analysis found that the amplitude of H2 was more negative than that of P2 in the right hemisphere (*p* < 0.05, *Cohen’s d* = 0.56).

In summary, in Session B, no significant difference was found among M, H1, and P1. Furthermore, H2 induced more negative N400 than P2.

## General Discussion

The present study aimed to explore how the meanings of ambiguous words interact with each other during L2 ambiguous word learning and how semantic similarity affects this learning process. The behavioral data showed that the learning outcome of the second meaning of polysemy (P2) was the best. The ERP data showed that the second meaning of homonym (H2) induced more negative N400 than the first meaning of homonym (H1), and P2 tended to induce more positive LPC in the frontal area than the first meaning of polysemy (P1) in session A. In session B, no significant difference was found among M, P1, and H1 in the N400. Furthermore, H2 induced more negative N400 than P2.

In session A, the N400 of H2 was more negative than that of H1, suggesting that the first meaning interferes with learning the second meaning of homonyms. As discussed in the introduction section, the amplitude of the N400 is related to the difficulty of semantic integration, that is, the more difficult a meaning gets integrated into semantic network, the larger the amplitude of the N400 becomes. Thus, the N400 of the second meaning of homonyms was more negative than that of the first meaning of homonyms, possibly due to a conflict between the two unrelated meanings which increases the difficulty to integrate the second meaning into the previously established semantic connection between the first meaning and the word form.

As for the polysemous words, the second meaning tended to induce more positive LPC (470–770 ms) than the first meaning in the frontal area. As discussed in the introduction section, the LPC may reflect the strength of memory, episodic memory retrieval and controlled semantic access. Johnson et al. (1998) found that LPC can be divided into three different subcomponents, and the memory process reflected by the parietal-occipital LPC is different from that reflected by the frontal LPC. The parietal-occipital LPC may reflect an explicit memory process, whereas the frontal LPC may reflect the retrieval of contextual information (Johnson et al., 1998). In the present study, P2 tended to induce more positive LPC relative to P1 in the frontal area, suggesting that learning the second meaning of polysemes may be facilitated by consciously retrieving the first meaning. Therefore, the more positive LPC in learning the second meaning of polysemes might be due to the semantic relatedness between the first and second meaning, which pre-activates the related semantic feature and increases the accessibility of the second meaning.

Based on the above results, we propose that there are both interference and facilitation during L2 ambiguous word learning. Specifically, the first meaning of homonyms learned earlier may interfere with the learning of a new second meaning. On the contrary, the first meaning of polysemes learned earlier may facilitate the learning of a new second meaning. Since the ERPs were collected while the participants learned the new meaning, these impacts from semantic similarity may happen during the encoding stage of the new meaning. Nevertheless, this does not necessarily rule out the possible impact of semantic similarity at the consolidation and retrieval stages of the new meaning.

The ERP data showed no significant N400 difference between the first meaning of ambiguous words and monosomy. This result seems to imply that learning the second meaning does not influence the first meaning, because if there exists any impacts from the second meaning on the first meaning, the N400 amplitudes of the first meaning of ambiguous words should be different from monosomies. However, our result showed no significant N400 difference between the first meaning of ambiguous words and monosomies. Therefore, we did not find evidence for the impact of the second meaning on the learning outcome of the first meaning in the present study. One explanation may be that primacy effect might exist when establishing the semantic representations of ambiguous words. Specifically, the representation of the first meaning of ambiguous words is more stable than that of the second meaning because the first meaning is learned early. Therefore, the representation establishment of the second meaning might be affected by the first meaning due to proactive impact, but the representation of the first meaning, which is more stable, is less likely to be affected by that of the second meaning, which is weaker. This finding is consistent with the results of Degani et al. (2014) which found that there was a clear advantage for the first-learned translation when multiple translations were learned on separate days. However, this does not mean that learning the second meaning will never impact the first meaning. The second meaning might affect the accessibility of the first meaning when the learning process is extended for a longer period of time. For example, recent studies found that learning new meanings impacts the accessibility of previously learned meanings of ambiguous words in both the second language (Zhang et al., 2020) and the first language (Fang et al., 2017). Therefore, it is possible that with the late learned meaning being increasingly consolidated, this late learned meaning might impact the retrieval of the first meaning.

The present study supports the model of ambiguous word processing which suggests that polysemes and homonyms are stored in different ways in the mental lexicon. Specifically, the multiple meanings of polysemes partially overlap, whereas the multiple meanings of homonyms are stored separately (Klepousniotou, 2002; Klepousniotou et al., 2012). Therefore, the competition among these multiple meanings delays the recognition of homonyms, which should lead to a homonym disadvantage. The overlap among these multiple meanings facilitates the recognition of polysemes, which should lead to a polyseme advantage. This is consistent with our results which showed an interference effect when learning the second meaning of homonyms and a facilitation effect when leaning the second meaning of polysemes. The behavioral result of the 4-to-1 forced choice task also showed that the learning performance of the second meaning of polysemes was better than that of the other four types of meaning, which further supports the predicted learning advantage for polysemous words.

In the present study, participants repeatedly learned the new meanings for six times in total. In future studies, we may manipulate the learning times to further explore the impact of learning intensity on the interaction mechanism among the multiple meanings of ambiguous words. Moreover, the participants we recruited were late unbalanced Chinese–English bilinguals with intermediate English proficiency. We may take a further step to explore the impact of L2 proficiency on ambiguous word learning in the future.

## Conclusion

To conclude, this was the first study to explore the interaction between two meanings in learning L2 ambiguous words and how semantic similarity affects this learning process. We found that the first meaning of homonyms interferes with learning the second meaning of homonyms, while the first meaning of polysemes facilitates the learning of the second meaning of polysemes. We did not find evidence that learning the second meaning impacts the first meaning learning. These results indicate that there are different mechanisms between learning L2 polysemes and homonyms. Establishment of the new meaning may be impaired by the semantic representation of the prior meaning when the new meaning is semantically unrelated to the prior meaning. However, when the new meaning is semantically related to the prior meaning, creating the new meaning representation may be facilitated by the semantic similarity between the two meanings.

## Data Availability Statement

The datasets generated for this study are available on request to the second author YL, zoe_cbje_236@163.com.

## Ethics Statement

The studies involving human participants were reviewed and approved by Ethical approval was obtained from the Committee of Protection of Subjects at Beijing Normal University. The patients/participants provided their written informed consent to participate in this study. The animal study was reviewed and approved by Ethical approval was obtained from the Committee of Protection of Subjects at Beijing Normal University.

## Author Contributions

YZ wrote the manuscript. YL collected and performed the data analysis. LL edited and revised the manuscript. BC designed the experiments and wrote the manuscript. All authors contributed to the article and approved the submitted version.

## Conflict of Interest

The authors declare that the research was conducted in the absence of any commercial or financial relationships that could be construed as a potential conflict of interest.
