# Supplementary material for: The Effect of Semantic Similarity on Learning Ambiguous Words in a Second Language: An Event-Related Potential Study
Source: Front Psychol. 2020 Jul 14;11:1633. doi: 10.3389/fpsyg.2020.01633 (PMC7381155; doi:10.3389/fpsyg.2020.01633)
Supplement: Supplementary file 1 [file Table_1.docx]

| Table S1. The polysemy learned in the learning task | | |
| --- | --- | --- |
| pseudowords | The First meaning | The Second meaning |
| soctur | 战争(WAR) | 士兵(SOLDIER) |
| osgion | 本领(ABILITY) | 技能(SKILL) |
| extow | 温和(MILD) | 阳光(SUNSHINE) |
| somius | 继承(SUCCEED) | 后代(HEIR) |
| pilects | 贫困(POVERTY) | 乞讨(BEG) |
| coggon | 讨厌(HATE) | 憎恶(ABHOR) |
| reourd | 照顾(CARE) | 帮助(HELP) |
| vussal | 预兆(HINT) | 趋势(TENDENCY) |
| conshats | 箭步(STRIDE) | 奔跑(WALK) |
| coupply | 律师(LAWYER) | 辩护(JUSTIFY) |
| mispray | 盼望(WISH) | 期待(HOPE) |
| kemble | 计算(COUNT) | 数学(MATHEMATICS) |
| oroun | 体育(GYMNASTICS) | 肌肉(MUSCLE) |
| commol | 天空(SKY) | 飞行(FLY) |
| purwase | 雾气(FOG) | 乌云(CLOUD) |
| euple | 指导(COACH) | 教书(TEACH) |
| lylen | 分离(SPLIT) | 撕毁(TEAR) |
| tadten | 主要(PRIMARY) | 核心(CORE) |
| cirfeit | 谷物(CEREAL) | 玉米(CORN) |
| jenoor | 对付(COPE) | 处理(DEAL) |
| rurker | 破晓(DAYBREAK) | 黎明(DAWN) |
| resahs | 辩论(DEBATE) | 争执(CONFLICT) |
| tabier | 困惑(CONFUSION) | 好奇(CURIOSITY) |
| matteft | 顾客(CUSTOMER) | 服务(SERVICE) |
| nawpew | 植物(PLANT) | 土壤(SOIL) |
| brirer | 货物(FREIGHT) | 运输(TRANSPORTATION) |
| wromid | 古董(ANTIQUE) | 陈旧(OLD) |
| goniln | 正直(INTEGRITY) | 忠诚(HONESTY) |
| rezell | 性格(PERSONALITY) | 品质(CHARACTER) |
| badpus | 鲸鱼(WHALE) | 巨大(GIANT) |
| cassus | 内疚(GUILT) | 后悔(REGRET) |
| odthaf | 放松(RELAXATION) | 休息(REST) |
| sanyac | 保护(GUARD) | 拯救(SAVE) |
| oumig | 棉花(COTTON) | 柔软(SOFT) |
| jeerfal | 美女(BEAUTY) | 漂亮(PRETTY) |
| Note：the English translation equivalents of Chinese words were listed in the brackets. | | |
