# Supplementary material for: The Effect of Semantic Similarity on Learning Ambiguous Words in a Second Language: An Event-Related Potential Study
Source: Front Psychol. 2020 Jul 14;11:1633. doi: 10.3389/fpsyg.2020.01633 (PMC7381155; doi:10.3389/fpsyg.2020.01633)
Supplement: Supplementary file 2 [file Table_2.docx]

| Table S2. The homonym learned in the learning task | | |
| --- | --- | --- |
| pseudowords | The First meaning | The Second meaning |
| zesho | 假设(HYPOTHESIS) | 悲痛(LAMENT) |
| nalpew | 地点(SITE) | 手段(METHOD) |
| storim | 饲料(PROVENDER) | 微弱(FAINT) |
| rebipt | 模仿(MIMIC) | 浪费(WASTE) |
| facure | 毛孔(PORE) | 伪装(CAMOUFLAGE) |
| desank | 发展(DEVELOPMENT) | 花粉(POLLEN) |
| kalleb | 嘲弄(RIDICULE) | 功能(FUNCTION) |
| rastion | 条理(ORDER) | 下巴(CHIN) |
| hullox | 详尽(COPIOUS) | 地板(FLOOR) |
| terfey | 驯化(TAME) | 包裹(PARCEL) |
| fugril | 明亮(LIGHT) | 饮水(DRINK) |
| effold | 坚固(FIRM) | 排除(ELIMINATION) |
| dumard | 警惕(WARY) | 理由(REASON) |
| visture | 排放(EMISSION) | 破坏(DAMAGE) |
| surberg | 供给(PROVISION) | 照片(PHOTOGRAPH) |
| stospept | 寓言(FABLE) | 注视(STARE) |
| canpit | 娱乐(ENTERTAIN) | 重力(GRAVITY) |
| witop | 斑点(MARK) | 掌握(MASTERY) |
| niotut | 海藻(SEAWEED) | 习俗(CUSTOM) |
| dessoll | 龙虾(LOBSTER) | 界限(BOUNDARY) |
| walage | 铃铛(BELL) | 皮肤(SKIN) |
| vasug | 感受(FEEL) | 垂直(VERTICAL) |
| rebunk | 面包(BREAD) | 评判(EVALUATE) |
| opber | 坚硬(RIGID) | 欺骗(CHEAT) |
| hortyr | 关闭(CLOSE) | 肤色(FUSE) |
| pullir | 经历(EXPERIENCE) | 红肿(WELT) |
| hidmet | 闪耀(SHINY) | 房屋(HOUSE) |
| yappab | 天赋(TALENT) | 代替(SUBSTITUTE) |
| welhase | 子弹(BULLET) | 农民(PEASANT) |
| eirlet | 简朴(PLAIN) | 逃跑(ESCAPE) |
| stovoy | 声称(CLAIM) | 加热(HEAT) |
| marark | 柴火(FIREWOOD) | 推荐(RECOMMEND) |
| cosurl | 画图(SKETCH) | 打击(PUNCH) |
| zelft | 开放(OPEN) | 砂砾(GRAVEL) |
| wroma | 俯冲(PLUNGE) | 文化(CULTURE) |
| Note：the English translation equivalents of Chinese words were listed in the brackets. | | |
